# Unmasking the Masquerader: A Delayed Diagnosis of MS and Its 4.5 Years of Implications in an Older African American Male

**DOI:** 10.1155/2019/5787206

**Published:** 2019-08-07

**Authors:** Deanna Dong, Joshua Carlson, Joseph Ruberwa, Thomas Snihur, Nawar Al-Obaidi, José Bustillo

**Affiliations:** ^1^St. George's University School of Medicine, St. George's, West Indies, Grenada; ^2^Newark Beth Israel Medical Center, Department of Internal Medicine, Newark, NJ, USA

## Abstract

Multiple sclerosis (MS) has classically been described as a disease of the young Caucasian female. While the prevalence may seem to be higher in Caucasians (CAs), recent studies suggest that the real incidence of MS may actually be higher in African Americans (AAs). Here, we discuss a nonclassical case of MS in an older African American male, prognostic factors, disease patterns in African Americans, and how a delay in diagnosis and socioeconomic factors can lead to worse outcomes. In patients that present with possible symptoms of MS, a high suspicion for MS should be entertained even in epidemiologically atypical patients to prevent delay in diagnosis and irreversible disability.

## 1. Introduction

Multiple sclerosis (MS) is an autoimmune, inflammatory demyelinating disease of the central nervous system affecting nearly 2 million people worldwide and approximately 400,000 in the United States alone [1–3]. MS can present with a wide variety of symptoms, from fatigue and cognitive difficulties to focal sensory and motor deficits. It may also present with bowel and urinary dysfunction, visual defects, and spasticity. MS is most commonly found in middle-aged women of European descent aged 20–50 years old [2, 3]. However, recent studies have reported a higher incidence of MS in African Americans compared to Caucasians, in addition to a 47% increased risk of MS in AAs when compared to CA counterparts [3–5].

When diagnosed in African Americans, MS has also been shown to have a more aggressive disease course compared to Caucasians and socioeconomic status can play a major role. This includes more frequent relapses, worse ambulatory dysfunction, more tissue damage, and a higher lesion burden in the CNS [3, 6–9]. A 2006 study of 21,557 patients from the NARCOMS registry of MS patients in North America showed that African Americans had increased odds of severe disease in multiple domains—hand, vision, cognition, and mobility—compared to Caucasians, and accounting for socioeconomic status reduced the strength of associations [3, 11]. This suggests that socioeconomic status is a confounding variable and is associated with increased disability. The severity of MS is often measured by the Expanded Disability Status Score (EDSS) or the Multiple Sclerosis Severity Scale (MSSS) [12, 13]. The MSSS is based on the EDSS with the addition of taking disease duration into account. A number of more recent studies show that African Americans with MS had a higher hazard ratio of reaching EDSS and MSSS milestones in less time [6, 7, 10, 14, 15].

Here, we discuss a case of an African American male who presented to the health system multiple times for upper and lower extremity weakness and urinary incontinence, who after 4.5 years of presenting with similar complaints was finally diagnosed with MS. Throughout that time period, he sought other means of pain and stiffness control, such as opioid analgesics, which should have been avoided with prompt recognition of the disease.

## 2. Case Presentation

A 53-year-old African American male, ex-railroad worker, presented with back pain and acute worsening of right arm and bilateral lower extremity weakness and spasticity, disabling him from moving from the bed to his rolling walker.

His past medical history consisted of a motor vehicle accident with open reduction internal fixation of the left ankle, heroin and cocaine use now on daily methadone, and chronic back pain, the MVA being a possible contributor. He had multiple admissions since March 2014, when he was 48 years old, for back and leg pain with lower extremity weakness. Imaging at the time showed extensive multilevel disc herniation, arthropathy, and foraminal stenosis in the cervical and lumbar regions. He reported self-medicating with oxycodone and sniffing heroin, as he stated it would alleviate muscle stiffness and pain and help him walk better. Over multiple ED visits, he was discharged on naproxen and advised to follow-up as an outpatient with a neurologist but was unable due to incarceration and lack of transportation after loss of driving capability. His weakness progressed from needing no assistance, to cane support, to rolling walker. On each presentation, the pain was described as sharp and stabbing, associated with intermittent numbness and tingling. Family history was only significant for hypertension. Social history significant for 30 pack-year tobacco use, substance abuse as mentioned, and occasional alcohol consumption. He had no allergies, and a review of systems was negative for current urinary/bowel changes, fevers, chills, weight loss, eye pain, or changes in appetite.

On physical exam, vital signs were T 36.8°C, HR 72 bpm, BP 140/89 mmHg, RR 18 breaths/min, SaO_2_ 99% on room air, and BMI 23.4. He was a tall, lean man, with a child-like demeanor and had difficulty processing, repeating, and retaining new terms. Pertinent findings demonstrated hypertonicity of muscles through right arm and bilaterally in thighs, calves, and ankles. Patellar and Achilles' reflexes were 3+ bilaterally, and right brachioradialis and triceps were 3+. Left upper extremity reflexes were 2+. He had a “swinging gait” where he would hold his walker and swing one leg, lock, and then swing the other leg. The remainder of his physical exam was unremarkable.

**Figure 1:**
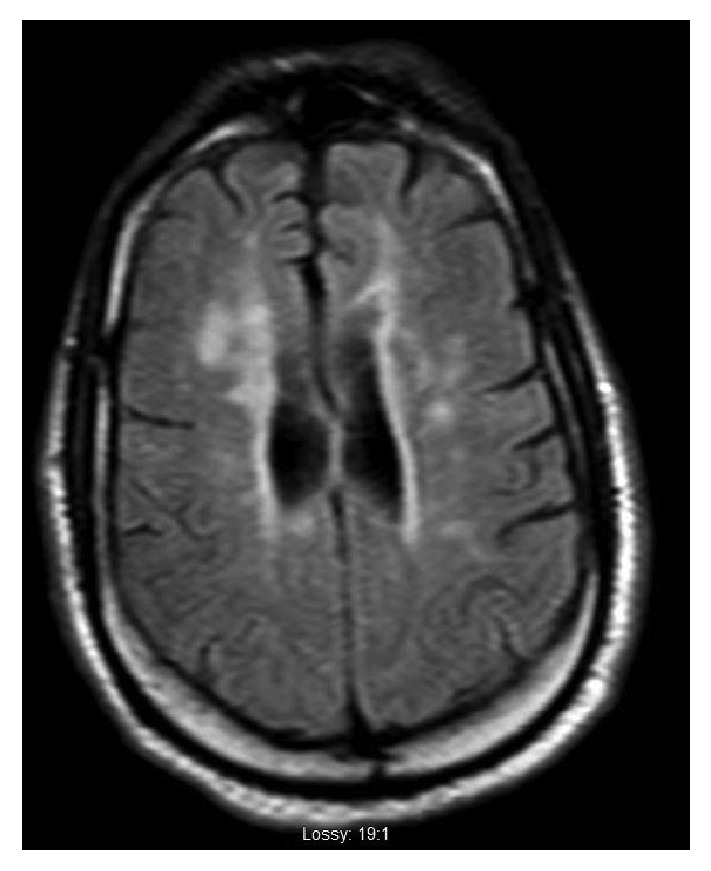
Abnormal hyperintense periventricular white matter lesions on MRI brain T2 FLAIR sequencing.

**Figure 2:**
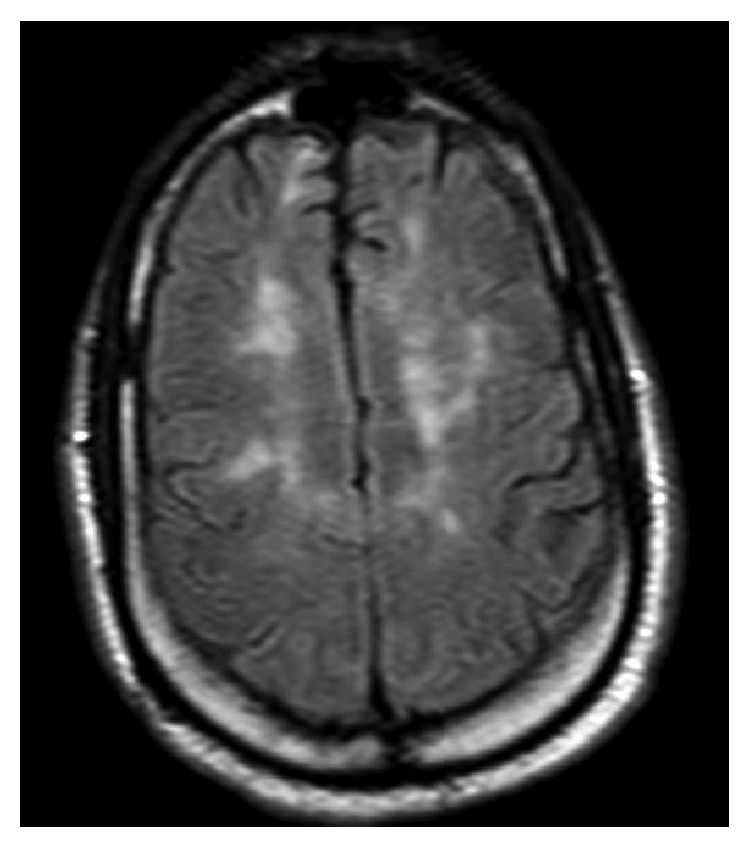
Additional subcortical hyperintensities on MRI brain T2 FLAIR sequencing.

**Figure 3:**
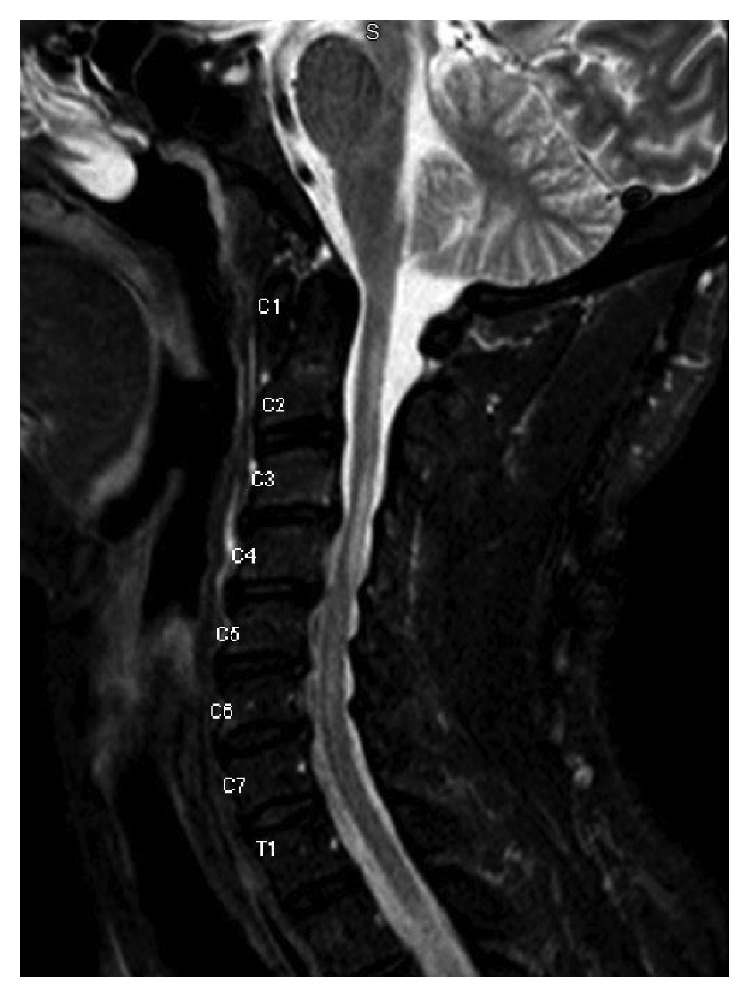
Hyperintense lesions representing demyelination from C2 to C6 on MRI C-Spine T2 FLAIR sequencing.

Lab work was unremarkable except for anemia with hemoglobin of 10.4 g/dL and borderline low calcium of 8.7 mg/dL. Prior autoimmune labs of ANA, RPR, anti-Scl 70, antihistone, anti-dsDNA, anti-Smith, anti-SSA/SSB, anti-RNP, and RH factor were all negative. The 25-hydroxy vit D level was low at 24 nmol/L. A CT lumbar spine showed similar findings to prior CT scans of facet joint arthropathy, foraminal stenosis, and nerve root compressions from L1 down. However, there was a new T2 hyperintense lesion at the conus medullaris at the T12/L1 level, measuring 1 cm in diameter, read as suggestive of demyelinating disease. A brain MRI showed similar areas of patchy white matter changes to prior brain MRI from April 2016, with an increased number of periventricular white matter changes that had abnormal diffusion on FLAIR signal, highly suggestive of demyelinating disease (Figures 1 and 2); however, no acute demyelination was deemed visible in the brain. A MRI cervical spine also showed abnormal T2 FLAIR enhancement from C2 to C6, suggestive of chronic demyelination (Figure 3). No optic nerve involvement was seen. A lumbar puncture was performed, with colorless CSF, with minimal RBC, WBC, and no evidence of infection. CSF myelin basic protein came back first, which was negative.

Using the McDonald criteria, he was diagnosed with MS based on clinical presentation and imaging findings, likely progressive-relapsing subtype based on the history. He was treated with 5 days of high-dose methylprednisolone, his symptoms drastically improved, and he was discharged with close follow-up to an MS specialist. After the patient's discharge, CSF IgG oligoclonal bands came back significantly positive, which were not seen in the patient's serum.

## 3. Discussion

Many past studies have found that MS is more common in women of North European descent, but recent studies have shown that the incidence of MS may actually be higher in African Americans and that individuals with African ancestry tend to have a more aggressive disease course with higher lesion burden [3, 5, 8, 14]. It is possible that the disease burden in African Americans is higher than it seems, as they tend to have a lower number of participants in large studies such as the NARCOMS registry [11]. However, a national study conducted in Brazil, in an ancestrally heterogeneous population, found that any African ancestry within the past three generations was associated with worse disease progression compared to patients without African ancestry [16]. AA patients, like our patient, are more likely than CA patients to have poor prognostic factors. We discuss the prognostic factors of MS and in our patient and how a delay in diagnosis led to a worse outcome.

In regard to this case, the African American male discussed presented several times since 2014 with symptoms that were not atypical for MS—flares associated with muscle weakness, spasticity, hypertonicity, and progressive decline in function. However, he was epidemiologically atypical as MS in African Americans versus Caucasians has an even higher female preponderance, about 80% versus 70% [4, 7, 16]. Additionally, his age of onset was greater than one standard deviation above the average age of symptom onset for AA patients and about two standard deviations over the mean age of onset for Caucasian patients. For this patient, there was a delay in proper diagnosis for over 4 years. A delay in diagnosis and proper treatment served to be particularly detrimental to this patient with numerous poor prognostic factors, including more aggressive disease course with faster progression in African Americans [3, 8].

The disease pattern of MS in AAs compared to CAs has higher rates of optic nerve impairment, older age at symptom onset, polysymptomatic onset, and transverse myelitis, among others, many of which are prognostic factors correlated with accelerated progression [3, 6, 7, 16]. In this case, the patient's poor prognostic factors of incomplete recovery after the initial acute attack, sphincter dysfunction, early relapse frequency, pyramidal and cerebellar symptoms at presentation, polysymptomatic presentation, motor symptoms, male gender, and older age at onset, correlated to an EDSS score of 6 out of 10, indicating accelerated disease progression [9, 16]. An interesting finding is that AAs are more likely to have opticospinal MS and transverse myelitis than CAs [6]. Our patient demonstrated transverse myelitis, which is still considered one of the poor prognostic factors.

In this case, as the patient's symptoms progressed, he found other means of self-medicating through the use of heroin and opioid analgesics obtained off the street to reduce spasticity and muscle pains and improve his mobility. The sequelae of his medical condition and his socioeconomic environment helped to fuel a psychological and physical dependence on opioid analgesics, leaving him with multiple problems.

Socioeconomic factors play a large role in the timing of diagnosis of MS and its proper management with lower socioeconomic status being linked to higher disability [11, 17]. It has been shown in the NARCOMS registry that African Americans typically had lower education levels, lower income, and lower coverage rates by private insurance compared to Caucasian Americans [11]. These factors could largely impact access to healthcare, disease understanding, and the ability to afford specific treatments. A study of Latinos, African Americans, and Caucasians showed that fewer African Americans (36.8%) were treated or evaluated at an MS center or clinic compared to 47% Latinos and 49.2% Caucasians [18]. In addition, African Americans, like the one presented in this case, may be underrepresented in this registry, as semiurban/urban minorities may never access appropriate care and be diagnosed with MS to even participate in the survey. While adjusting for SES factors reduced the strength of association between race and severity, it did not account for the entirety of the more severe disability found in AA patients [11].

In addition, Farber et al. hypothesized that since AAs are not the classic population for MS, there may be a higher number of emergency department visits between symptom onset to diagnosis time due to low suspicion [19]. But on the contrary, several studies have shown no significant increase or even decrease between time of first symptom onset and diagnosis between AA patients compared to CA patients [6, 7, 11, 14, 19]. In their retrospective study, Farber et al. found that race was not correlated to a higher number of ED visits or longer time to diagnosis, but faster time to diagnosis was associated with complaints of neurological symptoms. Given that a higher proportion of AA patients report neurological symptoms like sensory loss, paresthesia, vision problems, limb weakness, imbalance, and back pain, this could account for the equivalent and even shorter time periods to diagnosis found between AA and CA patients despite lower rates of access to healthcare in AA patients. These findings suggest that the difference in severity cannot be attributed to a delay in diagnosis and instead may be attributed to other factors related to ancestry. Several HLAs have recently been found to be associated with MS in AAs though it is still unclear what role they may play in disease pathogenesis [7].

It is also important to address that unconscious or conscious physician biases towards patients who are active drug users may impede the ability to properly assess the patient's symptoms. The symptoms may be perceived as not “real” and not part of a medical disease and instead just drug-seeking behavior. A small study on physician and patient attitudes in patients who were active drug users showed that providers feared being deceived by a drug-using patient. Providers specifically questioned if requests for opiates were medically indicated or solely addictive behavior [20]. Physicians in this study avoided addressing the patient's chief complaints and felt uncomfortable with this population [20]. It is possible in this situation that providers' unconscious or conscious biases of this patient's history of heroin and oxycodone use hindered them from accepting the patient's concerns of pain and spasticity as true medical complaints, again contributing to a delay in diagnosis. Given Farber et al.'s findings, the fact that the patient's neurological symptoms went unaddressed and there was inadequate pain control at discharge suggest that physician bias contributed to a delay in diagnosis [19].

Cree et al. found the median time to EDSS 6 to be 16 years in AAs versus 22 years for CAs in their patient population [7]. The estimated EDSS for our patient was about 6–6.5 with progression to this point over the span of only around 4.5 years. Our patient had numerous prognostic factors that suggested more rapid progression. As MS symptoms are only partially reversible even with treatment due to permanent axonal damage in the CNS, it is pertinent to initiate early treatment and maintain a high level of suspicion even in epidemiologically atypical patients to prevent irreversible disability. It is also imperative to be vigilant in patients with poor prognostic factors with the potential for accelerated decline as in the case of this patient.

## 4. Conclusion

Multiple sclerosis, a disease classically thought to be among young women of European descent, is now presenting with higher incidence in African Americans and tends to have a more progressive course with higher lesion burden in this population [3, 8, 14]. There are multiple socioeconomic factors, such as decreased access to healthcare that may delay a patient's presentation with MS to medical personnel. Due to the common perception that MS is most common in middle-aged females, it is possible that early diagnosis and treatment of MS can be missed, thus leading to progressive neurological decline and worse outcomes for the patient. It is important for clinicians to remain vigilant regarding the increasing incidence and severity of MS in the African American population and have a high index of suspicion in order to recognize the disease early, initiate treatment, and refer to an MS specialist. As healthcare providers, it is our moral responsibility to minimize and reduce healthcare disparities through recognition and awareness of personal bias, particularly in vulnerable populations as in our patient.
